# Serum sphingomyelin levels define oxyhemoglobin desaturation-related metabolic threshold in symptomatic obstructive sleep apnea

**DOI:** 10.1038/s41598-025-96386-9

**Published:** 2025-04-11

**Authors:** Ott Kiens, Egon Taalberg, Viktoria Ivanova, Ketlin Veeväli, Triin Laurits, Ragne Tamm, Aigar Ottas, Kalle Kilk, Ursel Soomets, Alan Altraja

**Affiliations:** 1 Department of Pulmonary Medicine, University of Tartu, Tartu, Estonia; 2 Lung Clinic, Tartu University Hospital, 167 Riia Street, Tartu, 50411 Estonia; 3 Institute of Biomedicine and Translational Medicine, University of Tartu, Tartu, Estonia; 4 Centre of Excellence for Genomics and Translational Medicine, University of Tartu, Tartu, Estonia; 5 Psychiatry Clinic, Tartu University Hospital, Tartu, Estonia

**Keywords:** Metabolome, Metabolism, Obstructive sleep apnea, Hypoxia, Metabolomic threshold, Metabolomics, Respiratory tract diseases

## Abstract

Hypoxia is a contributing factor for the morbidity and mortality in patients with obstructive sleep apnea (OSA). We aimed at identifying the percentage of sleep time with oxyhemoglobin desaturation below 90% (Tc90%) breakpoint from which the most significant changes occur in systemic metabolome of patients with OSA. In a prospective observational study on patients with polysomnography–confirmed symptomatic OSA, profiles of 186 metabolites including amino acids, biogenic amines, acylcarnitines (AC), lysophosphatidylcholines, phosphatidylcholines (PC) and sphingomyelins (SM) were analyzed with liquid chromatography-mass-spectrometry in peripheral blood, obtained at 3 time points that covered patients’ night sleep. Comparisons of rank-transformed data with general linear model for repeated measures after dichotomizing the study group at different Tc90% levels were applied to define the best cut-off, hypoxic metabolic threshold (HMT), based on Cohen’s f. Fifty-one subjects were recruited with their median Tc90% of 2.1. The mean Cohen’s f over the metabolites was highest (0.165) at a Tc90% of 1.8 representing the HMT. Of the different classes of metabolites, the Cohen’s f value at HMT was highest for SM (0.322). Compared to patients with Tc90% < HMT, concentrations of 2 PC, 1 AC and 7 SM were significantly higher in patients with Tc90% ≥HMT. The HMT for patients with OSA described in this report for the first time is located at a Tc90% level of 1.8 with SM levels contributing most to the size of this threshold.

**Supplementary Information:**

The online version contains supplementary material available at 10.1038/s41598-025-96386-9.

## Introduction

Obstructive sleep apnea (OSA) is characterized by the quantity of apneas and hypopneas per hour of sleep- apnea hypopnea index (AHI)- measured using night- time sleep studies^1^. OSA, when left untreated, augments the risk of cardiovascular disease^1,2^, motor vehicle accidents^3^and chronic airways disease exacerbations^4,5^. The classification of OSA into mild (AHI ⩾5 and < 15/h), moderate (AHI ⩾15 and < 30/h) and severe (AHI ≥ 30/h), using AHI^6^has recently received criticism^7^, because of different definitions used for diagnosing hypopnea^8^. Clinical severity of OSA is also influenced by other variables, such as cognitive function, presence of concomitant diseases, daytime sleepiness etc^8^. In some patient groups with sleep apnea, all-cause mortality has been better predicted by other parameters, such as the total time a patient had nocturnal oxyhemoglobin desaturation below 90% (T90)^9^. Higher percentage of sleep time with oxyhemoglobin desaturation below 90% (Tc90%) values have been associated with the risk of non-alcoholic steatohepatitis in OSA patients^10^. Both T90 and Tc90% thus reflect the hypoxic burden related to sleep-time breathing disorders and their higher values are related to unfavorable patient outcomes. Mainly the intermittent hypoxia, but also inflammatory processes and differences in oral microbiome drive metabolomic pathways of OSA^11,12^. Metabolomic studies on OSA patients have generally studied morning samples^13^, few studies have concentrated on the dynamic changes of the metabolome between multiple samples or night-time sampling^14^. Several studies have used untargeted analysis method^13^that is not reliably replicated among different laboratories^15^. Targeted analysis has shown better reproducibility between laboratories^16^.

We have previously thoroughly investigated the effects of apnea-hypopnea index on the metabolome^17^, however studies investigating the effects of hypoxia on OSA patients are currently lacking. According to our knowledge, there have been no studies assessing the Tc90% values from where the largest effect size on the metabolome takes place and which metabolite classes contribute most to hypoxia related metabolomic changes. The aim of our study was to find the Tc90% level in OSA patients where the metabolome changes are at their peak, a hypoxic metabolomic threshold (HMT) and to find which metabolites contribute most to these changes.

## Materials and methods

### Study subjects

Individuals of at least 18 years of age were recruited randomly from April 2018 until January of 2020 at the Department of Psychiatry of the Tartu University Hospital, which is our referral center for patients experiencing sleep-related issues. The subjects were required to have one of the following sleep-related complaint: insomnia symptoms, fatigue, sleepiness, waking up gasping, snoring or night-time breathing interruptions. Patients were referred to PSG mainly to confirm or exclude OSA or restless legs syndrome. The inclusion criteria were diagnosis of OSA based on symptoms and PSG findings. The following exclusion criteria were used: AHI < 5/h, treatment with continuous positive airway pressure during the last 6 months, any acute illness, defined as the presence of symptoms of acute infection, concomitant chronic illness such as heart failure in New York Heart Association class III-IV, chronic liver disease, autoimmune disease, degenerative cerebrovascular disease, chronic kidney disease stage IV-V, pulmonary disease with baseline oxyhemoglobin saturation levels below 93%, type I and type II diabetes, chronic neurological disease, active malignancy and treatment with drugs known to affect metabolome (systemic corticosteroids, antirheumatic drugs and hormonal contraceptives). The effect of different factors that can influence the body metabolome was minimized^18,19^: the patients were video-monitored, they did not eat during the study and stayed overnight in the same room under similar circumstances. STOP-BANG score results were recorded as per routine screening procedure for OSA^20^.

## Study design

This was a prospective observational study to assess the relationship between the peripheral blood metabolomic profiles and Tc90% in OSA patients referred for PSG. The study was done in accordance with the Declaration of Helsinki and the Tallinn Medical Research Ethics Committee approved the study protocol (decision number 2270). Each participant gave their written informed consent.

## Polysomnography

A standardized PSG recording^21^was performed in-hospital from 10 p.m. to 7:30 a.m. that included chin and leg electromyography, video monitoring, electroencephalography, electrooculography, nasal cannula, body position and snoring sensors, thoracoabdominal bands, heart rate and oxyhemoglobin saturation sensors as well as electrocardiography. Either an Embletta MPR (Natus Medical Inc., San Carlos, CA, USA) or a NOX A1 (Nox Medical, Reykjavik, Iceland) was used as PSG recording device. PSG data scoring was conducted partly manually (AHI values) and partly automatically (Tc90% values). The AHI values, which were based on obstructive respiratory events^22^, were obtained according to the guidelines of the American Academy of Sleep Medicine^21^.

## Blood sampling

Blood for the metabolome analyses was collected simultaneously with PSG recording via peripheral venipuncture on 3 different points of time: 9:00 p.m.; 5:00 a.m.; 7:00 a.m. These time points were selected for better characterization of metabolomic changes overnight on one hand and for minimally interfering with sleep on the other.

Serum samples were collected into BD Vacutainer silica-coated (REF 367614, Beckton Dickinson, Franklin Lakes, NJ, USA) extraction tubes. The samples were allowed to clot for 30 min at room temperature and were then centrifuged at 1,500 g for 15 min at 4 °C. Sera were consequently frozen at −80 °C until the conduction of further analysis. The process was completed within 60 min from venipuncture.

Common biochemistry analyses were conducted as per standard protocol (supplementary appendix). Metabolites present in the sera were analyzed using liquid chromatography-mass-spectrometry method. We used a targeted approach for assessing the levels of metabolites with previously validated AbsoluteIDQ p180 kit (BIOCRATES Life Sciences AG, Innsbruck, Austria). Concentrations of a total of 186 metabolites were measured: acylcarnitines, amino acids, biogenic amines, phosphatidylcholines (PC), lysophasphatidylcholines and sphingomyelins (SM) (for the full list of compounds refer to supplementary table S1). AbsoluteIDQ p180 kit is estimated to have inter-laboratory coefficients of variation below 10% for most metabolites used for the current study^16^. Sera were first thawed at room temperature and then analyzed with a QTRAP 4500 mass-spectrometer (Sciex, Framingham, MA, USA) that was connected to a high-performance liquid chromatography (Agilent 1260 series, Agilent Technologies, Waldbronn, Germany). Sample preparation and measurements were conducted as per manufacturer protocol in the test kit manual UM-P180, details of which have been previously described^23^. Metabolite concentrations were automatically calculated by the MetIDQ software (BIOCRATES Life Sciences AG). Deviations from quality control samples were evaluated separately and necessary corrections applied in METIDQ software previous to the data analysis. The metabolomic analysis was overall conducted identically to our previous work^14^.

### Data analysis

To find the minimum number of patients needed for this study, we relied on our pilot analyses on SM and determined that 48 individuals were sufficient to detect an effect size of ⩾0.31 (expressed as Cohen’s f)^24^ at 90% power and 5% two-sided significance level.

We used a ranked general linear model for repeated measures for detecting significant differences in the serum metabolite contents between populations that remained below and equal to or higher than the consecutively selected different Tc90% cut-off values. To accomplish this, all variables were rank transformed before the statistical analysis. Backward elimination of explanatory clinical and demographic variables was used to achieve the best fit for our model. In our final model, the outcomes were adjusted to the following covariates: age, body mass index (BMI), current smoking status, gender and serum contents of alanine aminotransferase, aspartate aminotransferase, high density lipoprotein cholesterol, low density lipoprotein (LDL) cholesterol, triglycerides, urea, potassium, and sodium. We used Fischer’s least significant difference method to correct for multiple comparisons. All the data obtained from analyzing the serum samples at 3 different time points via LC-MS was used in our model to detect significant differences in our study population. The measurements of 3 different time points were taken into account as separate measurements and analyzed statistically as triplicate.

The individuals with a Tc90% value below the HMT and those with a Tc90% value equal to or above the HMT were deemed to be below (HMT-) and above (HMT+) the HMT, respectively. Mann-Whitney U test and Pearson’s Chi Square test were used for comparing characteristics of the participants, as well as the participants below and at/above the HMT. The levels of specific metabolites and clinical parameters were correlated using Spearman correlation analysis. The data is presented as medians (interquartile ranges) or numbers (%). The IBM-SPSS software, version 20.0 (IBM Co, NY, USA) was used to perform the statistical analyses.

## Results

A total of 51 individuals (24 females and 27 males) were recruited with a median age of 57 (46.5–60) years. The median BMI of the study population was 29.7 (27.4–34.5) kg/m^2^ and median Tc90% was 2.1 (0.25–12.2) (Table 1).

**Table 1:** Baseline characteristics of the overall study population of individuals with symptomatic OSA: those with Tc90% below the hypoxic metabolomic threshold (Tc90% < 1.8) are termed as HMT-) and those with Tc90% above the hypoxic metabolomic threshold (Tc90% ≥ 1.8) are termed as HMT+).

Variable	Overall population (*n* = 51)	HMT- (*n* = 24)	HMT+ (*n* = 27)	*p*-value^#^
Age, years	57.0 (46.5–60.0)	47.5 (43.8–60.0)	57.0 (55.0–59.5)	0.053
BMI, kg/m^2^ Moderate-to-severe OSA, n (%)	29.7 (27.4–34.5) 33 (64,7)	27.5 (23.7–29.5) 13 (54.2)	34.2 (29.9–35.9) 20 (74.1)	< 0.001 0.23
Male gender, n (%)	27 (52.9)	15 (62.5)	12 (44.4)	0.31
Active smokers, n (%)	16 (31.4)	7 (29.2)	9 (33.3)	0.99
Neck circumference, cm	41.0 (39.0–43.0)	41.0 (39.0–43.0)	41.0 (39.3–43.5)	0.72
STOP BANG score	5.0 (4.0–6.0)	5.0 (3.0–5.0)	5.0 (4.0–6.0)	0.12
ESS score	8.0 (4.5–12.0)	9.5 (5.0–12.0)	8.0 (3.5–11.5)	0.55
Tc90% T90, min	2.1 (0.25–12.2) 9.2 (1.0–49.5)	0.15 (0.0–0.6) 0.7 (0.0–2.3)	12.1 (5.9–22.0) 49.0 (28.9–86.1)	< 0.001 < 0.001
AHI, events/h ODI 5%, events/h	19.5 (13.0–36.0) 1.8 (0.55–6.1)	16.9 (10.3–23.8) 0.95 (0.3–1.73)	30.7 (15.2–44.3) 4.1 (2–14.45)	0.012 < 0.001
ALAT, U/L	23.0 (19.5–29.5)	22.5 (15.5–28.3)	25.0 (21.0–33.5)	0.12
ASAT, U/L	22.0 (20.0–26.5)	22.5 (19.0–26.5)	22.0 (20.5–26.5)	0.79
FBG, mmol/L	5.8 (5.4–6.45)	5.65 (5.2–6.2)	5.9 (5.6–6.6)	0.079
LDL-Chol, mmol/L	3.40 (2.63–3.97)	3.44 (2.68–3.96)	3.13 (2.60–3.97)	0.74
HDL-Chol, mmol/L	1.10 (0.94–1.40)	1.21 (0.94–1.40)	1.06 (0.94–1.35)	0.95
TG, mmol/L	1.61 (1.11–2.34)	1.55 (0.98–1.98)	1.63 (1.31–2.48)	0.23

Data are presented as median (interquartile range) unless otherwise specified. ^#^: Comparisons were done using either Mann-Whitney U test (for continuous variables) or Pearson’s Chi square test (for categorical variables) and p-values are reported for differences between the MT- and MT + groups.

ALAT: alanine aminotransferase; ASAT: aspartate aminotransferase; BMI: body mass index; ESS: Epworth sleepiness scale; FBG: fasting blood glucose; HDL-Chol: high-density lipoprotein cholesterol; IQR: interquartile range; LDL-Chol: low-density lipoprotein cholesterol; ODI 5%: Oxygen desaturation index based on 5% oxygen desaturations; OSA: obstructive sleep apnea; Tc90%: percentage of sleep time with oxyhemoglobin desaturation below 90%; TG: triglycerides; T90: total sleep time a patient had oxyhemoglobin desaturation below 90%.

**Fig. 1:**
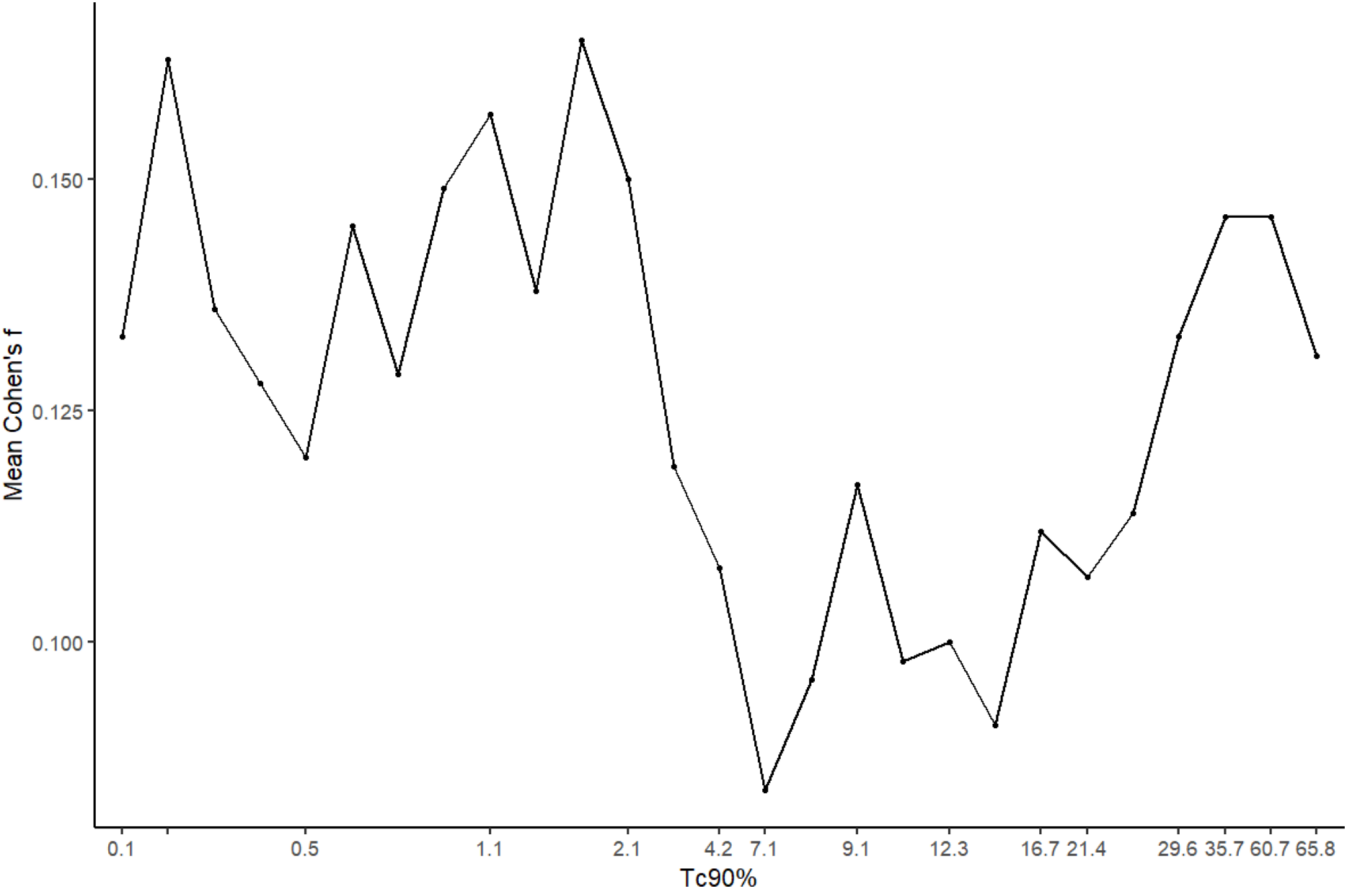
Average Cohen’s f values for different percentage of sleep time with oxyhemoglobin desaturation below 90% (Tc90%) thresholds in symptomatic patients with obstructive sleep apnea (*n* = 51). The average Cohen’s f value (0.165) was the highest at Tc90% of 1.8.

In our study population, the average Cohen’s f value of all the metabolites was highest at Tc90% of 1.8 (0.165) (Fig. 1). The exact Cohen’s f values together with the number of participants below and equal to or above the Tc90% threshold is presented in the supplementary table S2. This HMT by Tc90% divided the study population into 24 HMT- (47.1%) and 27 HMT + individuals (52.9%) (Table 1).

Compared to patients in the HMT- group, participants in the HMT + group were significantly more obese (*p* < 0.001) and had significantly higher AHI (*p* = 0.012), although they did not have a significantly larger neck circumference (Table 1). Also, patients in the HMT + group were older and had higher STOP-BANG scores, but these differences did not reach statistical significance (Table 1).

Of the different classes of metabolites, the Cohen’s f values at HMT were highest for SM (0.322) and amino acids (0.160) (Table 2). There were 10 metabolites that had significantly different concentrations between the HMT- and HMT + groups: two PC-s, one acylcarnitine and seven SM, all having heightened contents in the HMT + group (Table 3).

**Table 2:** Average Cohen’s F values by metabolomic substance classes at hypoxic metabolomic threshold (HMT) based on Tc90% value of 1.8 in symptomatic patients with obstructive sleep Apnoea (*n* = 51).

Metabolomic class	Average Cohen’s f value
Acylcarnitines Amino acids	0.141 0.160
Biogenic amines	0.106
Lysophosphatidylcholines	0.157
Phosphatidylcholines Sphingomyelins	0.155 0.322

**Table 3:** Metabolites with significantly different concentrations between the Sera of subjects with in symptomatic patients with obstructive sleep Apnoea (*n* = 51) with Tc90% below the hypoxic metabolomic threshold (Tc90% <1.8, termed as HMT-) (*n* = 24) And those with Tc90% above the metabolomic threshold (Tc90% ⩾ 1.8, termed as HMT+) (*n* = 27) at three time points: 9:00 P.m., 5:00 A.m. And 7:00 A.m.

Variable	Group	Time point			
		9:00 p.m.	5:00 a.m.	7:00 a.m.	p- value^#^
*Phosphatidylcholines*					
PC aa C38:5 PC ae C40:2	HMT- HMT+ HMT- HMT+	36.9 (31.3–43.8) 48.1 (38.0–52.3) 1.31 (0.89–1.56) 1.55 (1.36–1.71)	34.9 (25.9–40.9) 38.6 (29.8–46.4) 1.17 (0.89–1.37) 1.41 (1.18–1.61)	34.0 (27.7–38.3) 39.3 (30.7–44.6) 1.15 (0.93–1.39) 1.43 (1.31–1.56)	0.018 0.012
*Sphingomyelins*					
SM (OH) C22:1	HMT- HMT+	6.56 (4.79–8.69) 7.10 (5.46–8.17)	6.96 (3.80–8.90) 6.54 (4.82–7.67)	6.18 (4.55–8.24) 6.08 (4.89–7.30)	0.040
SM (OH) C22:2	HMT- HMT+	5.07 (4.11–7.49) 6.24 (5.08–7.99)	5.22 (3.46–7.49) 5.69 (4.12–6.96)	5.08 (3.68–6.92) 5.56 (4.29–6.97)	0.013
SM (OH) C24:1 SM C24:0 SM C24:1 SM C26:0 SM C26:1	HMT- HMT+ HMT- HMT+ HMT- HMT+ HMT- HMT+ HMT- HMT+	0.60 (0.48–0.77) 0.70 (0.55–0.78) 10.0 (8.4–12.9) 11.9 (8.7–13.0) 25.5 (19.6–35.4) 29.4 (24.5–34.6) 0.11 (0.078–0.147) 0.11 (0.091–0.145) 0.21 (0.15–0.29) 0.28 (0.20–0.33)	0.56 (0.44–0.80) 0.66 (0.52–0.76) 11.2 (7.4–13.4) 10.4 (8.3–12.5) 28.0 (17.9–33.7) 27.3 (22.4–32.2) 0.096 (0.073–0.14) 0.098 (0.078–0.14) 0.19 (0.16–0.27) 0.23 (0.19–0.34)	0.57 (0.42–0.78) 0.67 (0.53–0.73) 9.8 (7.6–12.9) 9.8 (8.4–11.6) 23.9 (19.9–33.1) 27.3 (22.2–32.0) 0.089 (0.077–0.14) 0.11 (0.082–0.14) 0.20 (0.19–0.23) 0.27 (0.21–0.30)	0.004 0.038 0.039 0.030 0.006
*Acylcarnitines*					
C16:1- OH	HMT- HMT+	0.016 (0.014–0.019) 0.018 (0.015–0.023)	0.018 (0.013–0.019) 0.018 (0.013–0.023)	0.016 (0.012–0.023) 0.017 (0.012–0.022)	0.037

Expressed as µmol/L (interquartile range). ^#^: Comparisons were done using ranked general linear model with repeated measures and p-values are reported for differences between the HMT- and HMT + groups.

PC aa C38:5: Phosphatidylcholine with diacyl residue sum C38:5; PC ae C40:2: Phosphatidylcholine with acyl-alkyl residue sum C40:2; SM (OH) C22:1: Hydroxysphingomyelin with acyl residue sum C22:1; SM (OH) C22:2: Hydroxysphingomyelin with acyl residue sum C22:2 ; SM (OH) C24:1: Hydroxysphingomyelin with acyl residue sum C24:1; SM C24:0: Sphingomyelin with acyl residue sum C24:0; SM C24:1: Sphingomyelin with acyl residue sum C24:1; SM C26:0: Sphingomyelin with acyl residue sum C26:0; SM C26:1: Sphingomyelin with acyl residue sum C26:1; C16:1-OH: Hydroxyhexadecenoylcarnitine (= Hydroxypalmitoleylcarnitine).

Significant time-dependent effects revealed by within-subject test were seen between HMT- and HMT + groups: two PC-s and one AC showed significantly different time-dependent effects (Supplementary Figure S1).

## Discussion

Our study showed that the average Cohen’s f value was highest at Tc90% of 1.8, thus denoting it as the HMT. Tc90% was not in a well-defined relationship with the overall serum metabolome (Fig. 1). Possibly different metabolic pathways turn to compensatory routes at different hypoxia thresholds, and there exist Tc90% ranges, where the metabolism remains unchanged despite hypoxia becoming gradually more severe. What is more, the Cohen’ s f value was mainly driven by higher effect sizes in SM, as judged upon both the particular Cohen’s f value for SM at the Tc90% level of 1.8, as well as the number of different SM with significant increase in comparison to that in HMT- patients. Assessment of different clinical variables could lead to better classification of moderate-severe OSA patients, as proposed previously^7^. HMT might thus be one parameter to be used in this classification.

Sphingolipids are a class of natural lipids composed of a sphingoid base foundation, sphingosine. Sphingolipids are important structural membrane lipids, as well as second messengers in diverse cellular signaling cascades^25^. When sphingosine is N-acylated with fatty acids, it forms ceramide^26^. Ceramide is a central molecule in sphingolipid biology^26^. When different moieties are attached to ceramide, more complex sphingolipids are formed^26^. SM are formed by attaching phosphoryl choline to ceramide^26^.

In our study, SM that had an acyl chain of C24:0 or C24:1 had concentrations significantly different between HMT + and HMT- populations (Table 3). It is known that plasma SM are an independent risk factor for coronary heart disease and it accumulates in human atheroma^27^. LDL cholesterol from atherosclerotic lesions contains more SM than LDL cholesterol from plasma^27^. Sphingomyelin synthase (SMS) isoforms 1 and 2 are involved in the last step of the sphingomyelin biosynthesis^27^. It has been shown that SMS1 and SMS2 overexpression increased the atherogenic potential of lipoproteins^27^, and demonstrated that SMs with C24:0 and C24:1 acyl chains are more associated with coronary artery disease^28^. SM (OH) C22:1 and SM C24:0 have been previously associated with an elevated risk of myocardial infarction^29^. Changes in HMT + OSA patient sphingomyelin levels are therefore probably associated with increased cardiovascular risk among these patients.

Relatively little information could be found regarding the role of hydroxylated sphingomyelins C22:2, C24:1, and sphingomyelins C26:0 and C26:1 regarding different disease processes. Future prospective studies in OSA patients would be needed to better clarify any associations between these sphingomyelins, OSA diagnosis and hypoxia.

 Altered metabolism of sphingolipids, including SM, has been linked to the development of obesity^30^, Type 1 and type 2 diabetes^31^. What is more, obesity is also linked to both night-time^32^and daytime^33^ hypoxia. In our study, the patients in HMT + group were more obese (Table 1), however the significant differences in the concentration of metabolites in Table 3 were calculated with a model that used BMI as a covariant, correcting for the effects that obesity might have had on the analysis.

Increased SM levels have also been associated with non-dipper hypertension in a study that used OSA as an exclusion criteria^30^. For in-depth description regarding the connection between OSA and non-dipper hypertension, we recommend the following review by Wolf et al.^34^. Briefly, non-dipper hypertension is associated with increased risk of cardiovascular events. Prevalence of non-dipper hypertension increases with OSA severity and effective treatment of OSA has been shown to improve nocturnal blood pressure control^34^. In our study, HMT + population had significantly higher AHI values (Table 1), however, as we did not measure the blood pressure values, it is difficult to assess, whether the increase in the level of SM is due to hypoxemia, non-dipper hypertension or some other unknown factor. Future studies with more specific data gathering are required to further specify the relationship between OSA, non-dipper hypertension and SM levels in peripheral blood.

In a large sample of heart failure patients with night-time sleep study data, it was shown that a T90 > 22 min but not the AHI value was associated with increased risk of mortality^9^. We have shown in our study that a Tc90% of 1.8 was the hypoxic metabolic threshold. Higher Tc90% values have been associated with the risk of non-alcoholic steatohepatitis in OSA patients^10^. Accumulating evidence shows that nocturnal hypoxemia is associated with important outcomes, e.g. increasing mortality, wide-spectrum metabolome changes and growing prevalence of concomitant diseases. However, no recommendations can be made for using a fixed nocturnal hypoxemia level to classify OSA patients because of scarce data.

Different phosphatidylcholines are usually synthesized via Kennedy pathway and phosphatidylcholine is one of the most abundant phospholipids of all mammalian cell membranes. Phospholipid metabolism can have numerous impacts in disease processes such as atherosclerosis, insulin resistance and obesity^35^. Future prospective studies in OSA patients would be needed to better clarify any associations between the phosphatidylcholines in Table 3, OSA diagnosis and hypoxia.

Acylcarnitines are fatty acid metabolites that play an important part in many cellular energy metabolism pathways. Acylcarnitines are increasingly being identified as important indicators in metabolic studies of many diseases, including metabolic disorders, cardiovascular diseases, diabetes, depression, neurologic disorders, and certain cancers^36^. In our study, statistically significant differences regarding C16:1-OH (Hydroxyhexadecenoylcarnitine) were found regarding HMT + and HMT- subjects. C16:1-OH has been previously demonstrated to be a potential biomarker for endometrial cancer^37^, its relationship regarding other diseases remains unknown. Future prospective studies in OSA patients would be needed to better clarify any associations between the acylcarnitine in Table 3, OSA diagnosis and hypoxia.

It has been previously shown that about 19% of the human metabolome demonstrates significant time-of-day variation. Metabolites showing significant 24-h variation have been shown to be from a variety of chemical classes and included acylcarnitines, lysophospholipids, bilirubin, corticosteroids, and amino acids. The mechanisms regulating this 24 h periodicity remain obscure but could be related to periodicity of metabolic processes such as gluconeogenesis, lipogenesis and tricarboxylic acid cycle^38^. In our study, we discovered that two PC- s and one AC show different time- dependent effects in the HMT + and HMT- group (supplementary table S1). We have previously demonstrated more changes in the overnight dynamics of OSA patients^14^. Hypoxia and OSA related mechanisms, such as excessive daytime sleepiness together with sleep fragmentation^39^ could be the possible mechanisms related to these time-dependent effects, but further studies are necessary to confirm this hypothesis.

The strengths of our study were assessment of the metabolome at 3 different time points, including night-time when the effect of OSA is presumably the highest, use of strict exclusion criteria and usage of venipuncture to obtain blood for the analyses. Indwelling catheters for blood sampling are associated with metabolome-affecting factors, such as heparin to flush the catheter and local inflammation caused by catheter itself^40^. Finally, recording of PSG and sampling of blood were done concurrently thus enabling more precise assessment of metabolome and sleep quality.

Limitations of our study included a relatively small sample size of 51 participants, even though the effect was neutralized by multiple sampling. The subjects in the HMT + group had higher BMI values. HMT + group had a higher percentage of OSA prevalence, albeit it did not reach statistical significance (Table 1). The measurement of hypoxic burden as the total area under the oxygen saturation curve from a pre- event baseline oxygen desaturation might be more accurate than using Tc90% as a proxy, albeit perhaps more difficult to implement^41^. However, there is still a lack of international widespread consensus on the gold standard of assessing hypoxia in OSA, since several parameters have been proposed for the assessment of hypoxic burden^42^. Therefore, future studies assessing the relationship of different hypoxic burden parameters to the metabolomic profile of OSA patients are needed. We found the effect size, based on Cohen’s f, of our study to be small, however it still enables, based on the original Cohen’s f interpretation^24^, to highlight the HMT. Thus, it cannot be deducted that the changes in the metabolome are insignificant nor that the shifts are biologically inconsequential. Lastly, it is important to emphasize that our findings are limited to symptomatic patients, because of our inclusion criteria.

## Conclusion

The hypoxic metabolomic threshold in OSA patients is located at Tc90% level of 1.8. Our study hence lends further support for the inclusion of hypoxic burden parameters for the classification of OSA.

## Electronic supplementary material

Below is the link to the electronic supplementary material.

Supplementary Material 1

## Data Availability

The datasets used and/or analyzed during the current study are available from the corresponding author on reasonable request.

## Declarations

### Support statement

This study was funded by the institutional research grant IUT 20–42 and the EU through the European Regional Development Fund (Project 2014-2020.4.01.15-0012) in addition to the Estonian Research Council grant (PUTJD914). The PSG studies done with Nox A1 polysomnograph were funded by ResMed.
