# Supplementary material for: Serum sphingomyelin levels define oxyhemoglobin desaturation-related metabolic threshold in symptomatic obstructive sleep apnea
Source: Sci Rep. 2025 Apr 11;15:12533. doi: 10.1038/s41598-025-96386-9 (PMC11992080; doi:10.1038/s41598-025-96386-9)
Supplement: Supplementary file 1 — Supplementary Material 1 [file 41598_2025_96386_MOESM1_ESM.pdf]

Supplementary Appendix

This appendix has been provided by the authors to give readers additional information about their work.

Supplement to: Serum sphingomyelin levels define oxyhemoglobin desaturation-related metabolic threshold in symptomatic obstructive sleep apnea

Authors: Ott Kiens[1,2], Egon Taalberg[3,4], Viktoria Ivanova[2], Ketlin Veeväli[5], Triin Laurits[5], Ragne Tamm[5], Aigar Ottas[3,4], Kalle Kilk[3,4], Ursel Soomets[3,4], Alan Altraja[1,2]

Institutions:

1. Department of Pulmonary Medicine, University of Tartu, Tartu, Estonia
2. Lung Clinic, Tartu University Hospital, Tartu, Estonia,
3. Institute of Biomedicine and Translational Medicine, University of Tartu, Tartu, Estonia
4. Centre of Excellence for Genomics and Translational Medicine, University of Tartu, Tartu, Estonia
5. Psychiatry Clinic, Tartu University Hospital, Tartu, Estonia

Corresponding Author: Ott Kiens, Lung Clinic, Tartu University Hospital, 167 Riia Street, 50411, Tartu, Estonia, ott.kiens@kliinikum.ee

General biochemical analyses

The serum levels of potassium, sodium, creatinine, urea, alanine aminotransferase (ALAT), aspartate aminotransferase (ASAT), triglycerides (TG), cholesterol, low-density lipoprotein cholesterol, and high-density lipoprotein cholesterol were measured once at 7:00 a.m. That of high-sensitivity C-reactive protein, haptoglobin, and ceruloplasmin levels were measured thrice at 9:00 p.m., 5:00 a.m. and 7:00 a.m. BD Vacutainer® Heparin (REF 368886, Beckton Dickinson, Franklin Lakes, NJ, USA) extraction tubes were used for the blood collection for the above-mentioned analyses.

Clinical biochemistry analyses were done at the United Laboratories of the Tartu University Hospital. Lipid profile (TG, cholesterol, low density lipoprotein, and high-density lipoprotein cholesterol) was analyzed via enzymatic colorimetric method. Concentrations of urea, ASAT, and ALAT were measured via kinetic photometric method. Potassium and sodium contents were analyzed using ion-selective electrodes. Creatinine concentration was obtained using an enzymatic method. High-sensitivity C-reactive protein, haptoglobin, and ceruloplasmin contents were all analyzed with use of immunoturbidimetric method. All these measurements were done fully automatically with a previously validated analyzer Cobas® 6000 c501 (Roche Diagnostics, F. Hoffmann-La Roche AG, Basle, Switzerland) [1].

Supplementary table S1. Full list of metabolites analyzed using the AbsoluteIDQ™ p180 kit (BIOCRATES Life Sciences AG, Innsbruck, Austria) with their abbreviations.

**Amino acids (n = 21)**	
**BC code**	**Analyte**
Ala	Alanine
Arg	Arginine
Asn	Asparagine
Asp	Aspartate
Cit	Citrulline
Gln	Glutamine
Glu	Glutamate
Gly	Glycine
His	Histidine
Ile	Isoleucine
Leu	Leucine
Lys	Lysine
Met	Methionine
Orn	Ornithine
Phe	Phenylalanine
Pro	Proline
Ser	Serine
Thr	Threonine
Trp	Tryptophan

Try	Tyrosine
Val	Valine
**Biogenic amines (n = 20)**	
Ac-Orn	Acetylornithine
ADMA	Asymmetric dimethylarginine
SDMA	Symmetric dimethylarginine
alpha-AAA	alpha-Aminoadipic acid
Histamine	Histamine
Met-SO	Methionine-Sulfoxide
Kyn	Kynurenine
Putrescine	Putrescine
Spermidine	Spermidine
Spermine	Spermine
Serotonin	Serotonin
PEA	Phenylethylamine
Nitro-Tyr	Nitrotyrosine
c4-OH-Pro	cis-4-Hydroxyproline
t4-OH-Pro	trans-4-Hydroxyproline
Creatinine	Creatinine
Carnosine	Carnosine
Taurine	Taurine
DOPA	Dihydroxyphenylalanine
Dopamine	Dopamine
**Acylcarnitines (n = 40)**	
C0	Carnitine (free)

C2	Acetylcarnitine
C3	Propionylcarnitine
C3:1	Propenoylcarnitine
C3-OH	Hydroxypropionylcarnitine
C4	Butyrylcarnitine/Isobutyrylcarnitine
C4:1	Butenoylcarnitine
C4-OH (C3-DC)	Hydroxybutyrylcarnitine (Malonylcarnitine)
C5	Isovalerylcarnitine/2-Methylbutyrylcarnitine/Valerylcarnitine
C5:1	Tiglylcarnitine/3-Methyl-crotonylcarnitine
C5:1-DC	Glutaconylcarnitine/Mesaconylcarnitine
C5-DC (C6-OH)	Glutarylcarnitine (Hydroxyhexanoylcarnitine (= Hydroxycaproylcarnitine))
C5-M-DC	Methylglutarylcarnitine
C5-OH (C3-DC-M)	Hydroxyisovalerylcarnitine/Hydroxy-2-methylbutyryl/Hydroxyvalerylcarnitine (Methylmalonylcarnitine)
C6 (C4:1-DC)	Hexanoylcarnitine (= Caproylcarnitine) (Fumarylcarnitine)
C6:1	Hexenoylcarnitine
C7-DC	Pimelylcarnitine
C8	Octanoylcarnitine (= Caprylylcarnitine)
C9	Nonanoylcarnitine (= Pelargonylcarnitine)
C10	Decanoylcarnitine (= Caprylcarnitine)
C10:1	Decenoylcarnitine
C10:2	Decadienoylcarnitine
C12	Dodecanoylcarnitine (= Laurylcarnitine)
C12:1	Dodecenoylcarnitine

C12-DC	Dodecanedioylcarnitine
C14	Tetradecanoylcarnitine (= Myristylcarnitine)
C14:1	Tetradecenoylcarnitine (= Myristoleylcarnitine)
C14:1-OH	Hydroxytetradecenoylcarnitine (= Hydroxymyristoleylcarnitine)
C14:2	Tetradecadienoylcarnitine
C14:2-OH	Hydroxytetradecadienoylcarnitine
C16	Hexadecanoylcarnitine (= Palmitoylcarnitine)
C16:1	Hexadecenoylcarnitine (= Palmitoleylcarnitine)
C16:1-OH	Hydroxyhexadecenoylcarnitine (= Hydroxypalmitoleylcarnitine)
C16:2	Hexadecadienoylcarnitine
C16:2-OH	Hydroxyhexadecadienoylcarnitine
C16-OH	Hydroxyhexadecanolycarnitine (= Hydroxypalmitoylcarnitine)
C18	Octadecanoylcarnitine (= Stearylcarnitine)
C18:1	Octadecenoylcarnitine (= Oleylcarnitine)
C18:1-OH	Hydroxyoctadecenoylcarnitine (= Hydroxyoleylcarnitine)
C18:2	Octadecadienoylcarnitine (= Linoleylcarnitine)
**Lysophosphatidylcholines (n = 14)**	
lysoPC a C14:0	Lysophosphatidylcholine with acyl residue C14:0
lysoPC a C16:0	Lysophosphatidylcholine with acyl residue C16:0
lysoPC a C16:1	Lysophosphatidylcholine with acyl residue C16:1
lysoPC a C17:0	Lysophosphatidylcholine with acyl residue C17:0
lysoPC a C18:0	Lysophosphatidylcholine with acyl residue C18:0
lysoPC a C18:1	Lysophosphatidylcholine with acyl residue C18:1
lysoPC a C18:2	Lysophosphatidylcholine with acyl residue C18:2
lysoPC a C20:3	Lysophosphatidylcholine with acyl residue C20:3

lysoPC a C20:4	Lysophosphatidylcholine with acyl residue C20:4
lysoPC a C24:0	Lysophosphatidylcholine with acyl residue C24:0
lysoPC a C26:0	Lysophosphatidylcholine with acyl residue C26:0
lysoPC a C26:1	Lysophosphatidylcholine with acyl residue C26:1
lysoPC a C28:0	Lysophosphatidylcholine with acyl residue C28:0
lysoPC a C28:1	Lysophosphatidylcholine with acyl residue C28:1
**Phosphatidylcholines (n = 76)**	
PC aa C24:0	Phosphatidylcholine with diacyl residue sum C24:0
PC aa C26:0	Phosphatidylcholine with diacyl residue sum C26:0
PC aa C28:1	Phosphatidylcholine with diacyl residue sum C28:1
PC aa C30:0	Phosphatidylcholine with diacyl residue sum C30:0
PC aa C30:2	Phosphatidylcholine with diacyl residue sum C30:2
PC aa C32:0	Phosphatidylcholine with diacyl residue sum C32:0
PC aa C32:1	Phosphatidylcholine with diacyl residue sum C32:1
PC aa C32:2	Phosphatidylcholine with diacyl residue sum C32:2
PC aa C32:3	Phosphatidylcholine with diacyl residue sum C32:3
PC aa C34:1	Phosphatidylcholine with diacyl residue sum C34:1
PC aa C34:2	Phosphatidylcholine with diacyl residue sum C34:2
PC aa C34:3	Phosphatidylcholine with diacyl residue sum C34:3
PC aa C34:4	Phosphatidylcholine with diacyl residue sum C34:4
PC aa C36:0	Phosphatidylcholine with diacyl residue sum C36:0
PC aa C36:1	Phosphatidylcholine with diacyl residue sum C36:1
PC aa C36:2	Phosphatidylcholine with diacyl residue sum C36:2
PC aa C36:3	Phosphatidylcholine with diacyl residue sum C36:3
PC aa C36:4	Phosphatidylcholine with diacyl residue sum C36:4

PC aa C36:5	Phosphatidylcholine with diacyl residue sum C36:5
PC aa C36:6	Phosphatidylcholine with diacyl residue sum C36:6
PC aa C38:0	Phosphatidylcholine with diacyl residue sum C38:0
PC aa C38:1	Phosphatidylcholine with diacyl residue sum C38:1
PC aa C38:3	Phosphatidylcholine with diacyl residue sum C38:3
PC aa C38:4	Phosphatidylcholine with diacyl residue sum C38:4
PC aa C38:5	Phosphatidylcholine with diacyl residue sum C38:5
PC aa C38:6	Phosphatidylcholine with diacyl residue sum C38:6
PC aa C40:1	Phosphatidylcholine with diacyl residue sum C40:1
PC aa C40:2	Phosphatidylcholine with diacyl residue sum C40:2
PC aa C40:3	Phosphatidylcholine with diacyl residue sum C40:3
PC aa C40:4	Phosphatidylcholine with diacyl residue sum C40:4
PC aa C40:5	Phosphatidylcholine with diacyl residue sum C40:5
PC aa C40:6	Phosphatidylcholine with diacyl residue sum C40:6
PC aa C42:0	Phosphatidylcholine with diacyl residue sum C42:0
PC aa C42:1	Phosphatidylcholine with diacyl residue sum C42:1
PC aa C42:2	Phosphatidylcholine with diacyl residue sum C42:2
PC aa C42:4	Phosphatidylcholine with diacyl residue sum C42:4
PC aa C42:5	Phosphatidylcholine with diacyl residue sum C42:5
PC aa C42:6	Phosphatidylcholine with diacyl residue sum C42:6
PC ae C30:0	Phosphatidylcholine with acyl-alkyl residue sum C30:0
PC ae C30:1	Phosphatidylcholine with acyl-alkyl residue sum C30:1
PC ae C30:2	Phosphatidylcholine with acyl-alkyl residue sum C30:2
PC ae C32:1	Phosphatidylcholine with acyl-alkyl residue sum C32:1
PC ae C32:2	Phosphatidylcholine with acyl-alkyl residue sum C32:2

PC ae C34:0	Phosphatidylcholine with acyl-alkyl residue sum C34:0
PC ae C34:1	Phosphatidylcholine with acyl-alkyl residue sum C34:1
PC ae C34:2	Phosphatidylcholine with acyl-alkyl residue sum C34:2
PC ae C34:3	Phosphatidylcholine with acyl-alkyl residue sum C34:3
PC ae C36:0	Phosphatidylcholine with acyl-alkyl residue sum C36:0
PC ae C36:1	Phosphatidylcholine with acyl-alkyl residue sum C36:1
PC ae C36:2	Phosphatidylcholine with acyl-alkyl residue sum C36:2
PC ae C36:3	Phosphatidylcholine with acyl-alkyl residue sum C36:3
PC ae C36:4	Phosphatidylcholine with acyl-alkyl residue sum C36:4
PC ae C36:5	Phosphatidylcholine with acyl-alkyl residue sum C36:5
PC ae C38:0	Phosphatidylcholine with acyl-alkyl residue sum C38:0
PC ae C38:1	Phosphatidylcholine with acyl-alkyl residue sum C38:1
PC ae C38:2	Phosphatidylcholine with acyl-alkyl residue sum C38:2
PC ae C38:3	Phosphatidylcholine with acyl-alkyl residue sum C38:3
PC ae C38:4	Phosphatidylcholine with acyl-alkyl residue sum C38:4
PC ae C38:5	Phosphatidylcholine with acyl-alkyl residue sum C38:5
PC ae C38:6	Phosphatidylcholine with acyl-alkyl residue sum C38:6
PC ae C40:1	Phosphatidylcholine with acyl-alkyl residue sum C40:1
PC ae C40:2	Phosphatidylcholine with acyl-alkyl residue sum C40:2
PC ae C40:3	Phosphatidylcholine with acyl-alkyl residue sum C40:3
PC ae C40:4	Phosphatidylcholine with acyl-alkyl residue sum C40:4
PC ae C40:5	Phosphatidylcholine with acyl-alkyl residue sum C40:5
PC ae C40:6	Phosphatidylcholine with acyl-alkyl residue sum C40:6
PC ae C42:0	Phosphatidylcholine with acyl-alkyl residue sum C42:0
PC ae C42:1	Phosphatidylcholine with acyl-alkyl residue sum C42:1

PC ae C42:2	Phosphatidylcholine with acyl-alkyl residue sum C42:2
PC ae C42:3	Phosphatidylcholine with acyl-alkyl residue sum C42:3
PC ae C42:4	Phosphatidylcholine with acyl-alkyl residue sum C42:4
PC ae C42:5	Phosphatidylcholine with acyl-alkyl residue sum C42:5
PC ae C44:3	Phosphatidylcholine with acyl-alkyl residue sum C44:3
PC ae C44:4	Phosphatidylcholine with acyl-alkyl residue sum C44:4
PC ae C44:5	Phosphatidylcholine with acyl-alkyl residue sum C44:5
PC ae C44:6	Phosphatidylcholine with acyl-alkyl residue sum C44:6
**Sphingomyelins (n = 15)**	
SM (OH) C14:1	Hydroxysphingomyelin with acyl residue sum C14:1
SM (OH) C16:1	Hydroxysphingomyelin with acyl residue sum C16:1
SM (OH) C22:1	Hydroxysphingomyelin with acyl residue sum C22:1
SM (OH) C22:2	Hydroxysphingomyelin with acyl residue sum C22:2
SM (OH) C24:1	Hydroxysphingomyelin with acyl residue sum C24:1
SM C16:0	Sphingomyelin with acyl residue sum C16:0
SM C16:1	Sphingomyelin with acyl residue sum C16:1
SM C18:0	Sphingomyelin with acyl residue sum C18:0
SM C18:1	Sphingomyelin with acyl residue sum C18:1
SM C20:2	Sphingomyelin with acyl residue sum C20:2
SM C22:3	Sphingomyelin with acyl residue sum C22:3
SM C24:0	Sphingomyelin with acyl residue sum C24:0
SM C24:1	Sphingomyelin with acyl residue sum C24:1
SM C26:0	Sphingomyelin with acyl residue sum C26:0
SM C26:1	Sphingomyelin with acyl residue sum C26:1

Supplementary Table S2. Average Cohen's f values for the different Tc90% thresholds and the numbers of participants with their Tc90% values below the Tc90% threshold and equal to or above the Tc90% threshold. At the Tc90% threshold of zero, the "No of participants < Tc90% threshold" had a Tc90% of exactly zero and the „No of participants ≥ Tc90% threshold" had Tc90% values above zero.

Tc90% threshold (% of sleep time)	Cohen's f	No of participants < Tc90% threshold	No of participants ≥ Tc90% threshold
0.1	0.105	21	44
0.2	0.113	23	42
0.3	0.095	24	41
0.4	0.095	26	39
0.5	0.080	27	38
0.6	0.103	28	37
0.7	0.101	30	35
0.8	0.118	31	34
1.1	0.132	33	32
1.6	0.120	34	31
1.8	0.129	36	29
2.1	0.113	37	28
3.4	0.096	39	26
4.2	0.089	41	24
7.1	0.072	43	22
8.5	0.081	45	20
9.1	0.095	47	18
10.6	0.091	49	16

12.3	0.096	51	14
13.5	0.083	53	12
16.7	0.121	55	10
21.4	0.108	57	8
22.5	0.109	58	7
29.6	0.116	61	4
35.7	0.131	62	3
60.7	0.128	63	2
65.8	0.123	64	1

Tc90%, percentage of sleep time with oxyhemoglobin saturation below 90%

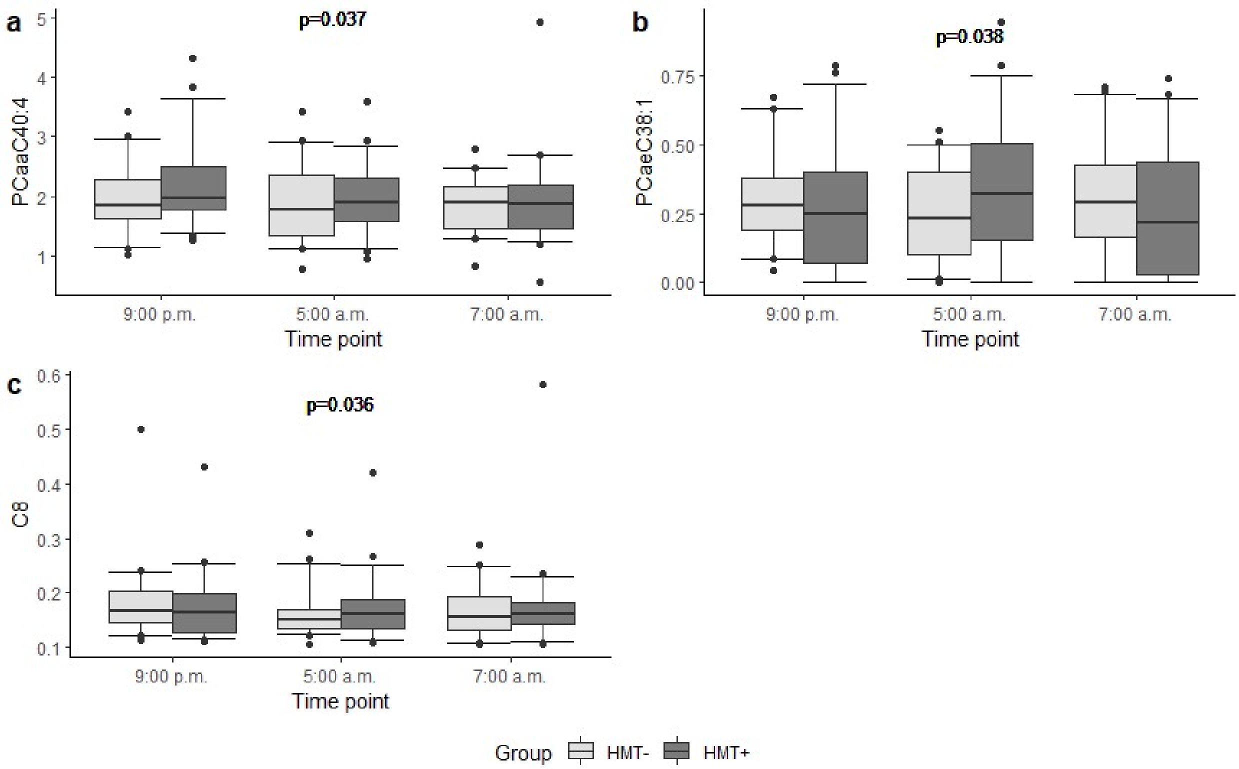

Supplementary Figure S1. Significant time-dependent effects unraveled by within-subject tests were seen in 2 phosphatidylcholines and 1 acylcarnitine pointing to significantly different overnight dynamics of these metabolites in the HMT- (below the hypoxic metabolomic threshold) and HMT+ (equal to or above the hypoxic metabolomic threshold) groups: a – PCaaC40:4: phosphatidylcholine with diacyl residue sum C40:4, b – PCaeC38:1: phosphatidylcholine with acyl-alkyl residue sum C38:1, c – C8: octanoylcarnitine (= caprylylcarnitine)

References

1. Supak Smolcic V, Bilic-Zulle L, Fisic E. Validation of methods performance for routine biochemistry analytes at Cobas 6000 analyzer series module c501. *Biochem Med (Zagreb)* 2011;21(2):182-90. doi: 10.11613/bm.2011.028 [published Online First: 2011/12/06]
